# SPECT and PET radiopharmaceuticals for molecular imaging of apoptosis: from bench to clinic

**DOI:** 10.18632/oncotarget.14730

**Published:** 2017-01-18

**Authors:** Xiaobo Wang, Han Feng, Shichao Zhao, Junling Xu, Xinyu Wu, Jing Cui, Ying Zhang, Yuhua Qin, Zhiguo Liu, Tang Gao, Yongju Gao, Wenbin Zeng

**Affiliations:** ^1^ Department of Nuclear Medicine, Henan Provincial People’s Hospital and People’s Hospital of Zhengzhou University, Zhengzhou, China; ^2^ Xiangya School of Pharmaceutical Sciences and Molecular Imaging Research Center, Central South University, Changsha, China; ^3^ Department of Pharmacy, Henan Provincial People’s Hospital and People’s Hospital of Zhengzhou University, Zhengzhou, China; ^4^ Department of Cardiology, Henan Provincial People’s Hospital and People’s Hospital of Zhengzhou University, Zhengzhou, China; ^5^ Department of Nuclear Medicine, Shandong Cancer Hospital and Institute, Shandong University, Jinan, China

**Keywords:** SPECT, PET, radiopharmaceuticals, apoptosis, clinical status

## Abstract

Owing to the central role of apoptosis in many human diseases and the wide-spread application of apoptosis-based therapeutics, molecular imaging of apoptosis in clinical practice is of great interest for clinicians, and holds great promises. Based on the well-defined biochemical changes for apoptosis, a rich assortment of probes and approaches have been developed for molecular imaging of apoptosis with various imaging modalities. Among these imaging techniques, nuclear imaging (including single photon emission computed tomography and positron emission tomography) remains the premier clinical method owing to their high specificity and sensitivity. Therefore, the corresponding radiopharmaceuticals have been a major focus, and some of them like ^99m^Tc-Annexin V, ^18^F-ML-10, ^18^F-CP18, and ^18^F-ICMT-11 are currently under clinical investigations in Phase I/II or Phase II/III clinical trials on a wide scope of diseases. In this review, we summarize these radiopharmaceuticals that have been widely used in clinical trials and elaborate them in terms of radiosynthesis, pharmacokinetics and dosimetry, and their applications in different clinical stages. We also explore the unique features required to qualify a desirable radiopharmaceutical for imaging apoptosis in clinical practice. Particularly, a perspective of the impact of these clinical efforts, namely, apoptosis imaging as predictive and prognostic markers, early-response indicators and surrogate endpoints, is also the highlight of this review.

## INTRODUCTION

*Nothing can happen more beautiful than death*.

-Walt Whitman

From the perspectives of a clinician, many diseases are associated with dysregulation of apoptosis (either too much or too little, respectively). Diseases such as myocardial infarction, neurodegenerative disorders (e.g. Parkinson and Alzheimers), transplant rejection are all characterized by too much apoptosis [1–3]. Conversely, cancer is often characterized by too little apoptosis and the capacity to evade apoptosis is known as one of the hallmark of cancer and associated with cancer development [4]. Therefore, current therapies of these diseases are also designed to prevent or induce cell apoptosis directly or indirectly [5, 6]. At present, histological methods such as terminal deoxynucleotidyl transferase (TdT) mediated dUTP-biotin nick end labeling (TUNEL) assays represent “gold standard” for detecting apoptosis in clinical settings [7–9]. However, these are primarily *in vitro* or *ex vivo* methods and are thus invasive, time consuming, and set limitations to many follow-up studies. The ongoing efforts for more sophisticated *in vivo* methods are needed and greatly expanding the collective understanding of apoptosis in living cells, experimental animals and, ultimately, humans.

Specific biochemical changes that occur in apoptotic cells or tissues may offer potential biomarkers for molecular imaging of apoptosis [10, 11]. Several of these molecular alterations that have been investigated to date, include phosphatidylserine exposed at the outer leaflet of plasma membrane, detected by proteins or peptides such as Annexin V, activated caspases (cysteine aspartyl specific proteases) in the intracellular compartment, detected by labeled enzyme substrates or inhibitors, apoptotic membrane imprint, detected by a set of novel small-molecule probes such as ApoSense family, DFNSH and DNSBA, and the collapse of mitochondrial membrane potential, detected by reduced levels of phosphonium cations that normally accumulate in healthy mitochondria [12].

Based on the well-defined biochemical changes, a rich assortment of probes and approaches have been developed for molecular imaging of apoptosis in preclinical and clinical studies, and documented in many reviews [11–18]. Majority of these probes have been developed for various imaging modalities such as single photon emission computed tomography (SPECT), positron emission tomography (PET), magnetic resonance imaging (MRI), optical imaging, ultrasound (US) and dual or multiple modality techniques. Although the spatial and temporal resolutions of SPECT and PET may not be as exquisite as some other imaging modalities, their specificity and sensitivity are impressive, thus making them more attractive for clinical applications [19]. Correspondingly, several radiopharmaceuticals including ^99m^Tc-Annexin V, ^18^F-ML-10, ^18^F-CP18, ^18^F-ICMT-11 have been developed and are currently under clinical investigations in Phase I/II or Phase II/III clinical trials. In this review, we focus mainly on these radiopharmaceuticals that have been widely used in clinical trials and elaborate them in terms of radiosynthesis, pharmacokinetics and dosimetry, and their applications in different clinical stages. We also explore the unique features required to qualify a desirable radiopharmaceutical for imaging apoptosis in clinical practice. Particularly, a perspective of the impact of these clinical efforts, namely, apoptosis imaging as predictive and prognostic markers, early-response indicators and surrogate endpoints, is also the highlight of this review.

## CLINICAL PERSPECTIVE OF APOPTOSIS IMAGING

In the clinical context, the interest of apoptosis imaging mainly focuses on early detection of disease, monitoring of disease course, assessment of treatment efficacy, or development of new therapies. Despite over decades of intense investigation, there are few routine clinical practices for molecular imaging of apoptosis in patients. This could be ascribed to the complex set of features required for a radiopharmaceutical with potentials for clinical molecular imaging of apoptosis [20]. Overall, a desirable radiopharmaceutical for imaging of apoptosis on a wide scope of diseases in clinical practice is expected to have the following unique features: 1) The radiopharmaceuticals should have high specificity and selectivity for apoptotic cells, detect the early stage of apoptosis, and preferably distinguish apoptosis from other types of programmed cell death such as programmed necrosis and autophagy; 2) To monitor biochemical process of disease at early stage, frequently requires spying on a very small amount of apoptotic cells. The radiopharmaceuticals, therefore, should manifest high specific activity so as to obtain optimal images; 3) The radiopharmaceuticals should have suitable *in vivo* properties such as favorable target-to-background ratio, adequate biodistribution, rapid distribution throughout the body, and rapid clearance from non-target organs; 4) The radiopharmaceuticals should have stability *in vivo* with minimal metabolism; 5) The radiopharmaceuticals should have minimal or acceptable level of immunogenicity and toxicity; 6) The radiopharmaceuticals should be possible to be produced in a Good Manufacturing Practices (GMP) grade synthesis system for the release of radiopharmaceuticals preparation for human applications. However, it would be even better to have a kit-based synthesis as this is more robust and easier to distribute globally; 7) The radiopharmaceuticals should be compatible with SPECT or PET imaging modality [21, 22]. In addition, timing is essential in imaging apoptosis in order to achieve a high-quality image, as apoptosis is a time-dependent process which is highly dependent on the specifics of the apoptotic phenomenon under study (e.g. types of tumor, chemotherapy) [11]. In consequence, it remains highly challenging for scientists to design and develop desirable radiopharmaceuticals for clinical use.

## RADIOPHARMACEUTICAL AND CLINICAL TRIALS

To date, a couple of radiopharmaceuticals have preliminary met these challenges and have advanced in Phase I/II or Phase II/III clinical trials. Table 1 summarizes these radiopharmaceuticals and their clinical applications in which SPECT and PET imaging of apoptosis has been interrogated in patients. These clinical trials show promise for clinical imaging of apoptosis in a variety of diseases.

**Table 1:** Overview of radiopharmaceuticals and their clinical applications on a wide scope of diseases

Radio- pharmaceuticals	Clinical Status	Medical Field	Clinical Applications	Ref
^99m^Tc-Annexin V	Phase II/III	Oncology	Diagnosis of native apoptosis in tumors	[23, 24]
			Early assessment of response to therapy	[25–27]
			Prognosis of overall or progression-free survival	[28]
		Cardiology	Assessment of cardiac infarction /reperfusion damage	[29, 30]
			Early diagnosis of heart failure	[31]
			Identification of unstable atherosclerotic plaque	[32]
		Neurology	Diagnosis of acute stroke and assessment of response to therapy	[33, 34]
			Diagnosis of AD in patients	[35]
		Organ Transplantation	Cardiac allograft rejection	[36, 37]
		Gastroenterology	Prediction of efficacy of therapy in Crohn's disease	[38]
		Ophthalmology	Detection of apoptotic retinal cells in glaucoma	[39]
		Orthopaedics	Differential identification of loosening and infection of prostheses	[40]
^18^F-ML-10	Phase II	Oncology	Early assessment of brain metastases to WBRT	[41]
			Apoptosis change in GBM early after therapy	[42]
^18^F-CP18	Phase I/II	Healthy Volunteers	Biodistribution and dosimetry	[43]
^18^F-ICMT-11	Phase I	Healthy Volunteers	Biodistribution and dosimetry	[44]
^123^I-Annexin V	Phase I	Healthy Volunteers	Biodistribution and dosimetry	[45]

### ^99m^Tc-Annexin V SPECT imaging of apoptosis

Annexin V is a small 35-36 kD calcium-dependent protein with a potent high-affinity for phosphatidylserine (Kd = 0.1 nM) [46]. Usually, phosphatidylserine is located in the inner membrane leaflet and not available for Annexin V binding. However, upon induction of apoptosis, it is externalized and remains on the outer leaflet of the membrane throughout the process, where it can bind with Annexin V in a Ca^2+^-dependent manner. Therefore, Annexin V is suitable for imaging of apoptosis. By coupling different radioactive isotopes to the Annexin V molecule, visualization of phosphatidylserine *in vivo* in animal models or even in patients using SPECT and PET has been performed and reviewed in these literatures [47–51]. Among them, ^99m^Tc-Annexin V is by far the most extensively investigated and broadly used apoptosis-detecting radiopharmaceutical to date.

Radiosynthesis. A variety of ^99m^Tc-Annexin V radiopharmaceuticals have been developed by different groups using various types of chelators and co-ligands, each resulting in a different biological behavior. ^99m^Tc-4,5-bis-(thioacetamido)pentanoyl-Annexin V (^99m^Tc-BTAP-Annexin V or ^99m^Tc-Apomate) was the first ^99m^Tc-Annexin V radiopharmaceutical to be described and evaluated in humans. This radiopharmaceutical was achieved by the pre-formed chelate approach in which an activated diamide dimercaptide N_2_S_2_ chelate was used, based on the OncoTrac labelling method [52], resulting in 25-30 % overall radiochemical yields and a specific activity of 58.3 GBq/μmol [53]. Subsequently, the production of ^99m^Tc-BTAP-Annexin V was further optimized in a kit formulation (Apomate, Theseus Imaging Corporation, Boston, USA). However, this ^99m^Tc-BTAP-Annexin V kit formulation has elaborate and time-consuming procedures (about 75 min, high start activities of ^99m^Tc) but relatively low radiochemical yields. Therefore, an improved ^99m^Tc-Annexin V radiopharmaceutical is needed. As an alternative radiolabelling approach for Annexin V, the preparation of ^99m^Tc-hydrazinonicotinamide-Annexin V (^99m^Tc-HYNIC-Annexin V) was firstly proposed by the group of Blankenberg *et al*. in 1998 using the HYNIC technology [54], which was originally developed by Abrams [55]. The hydrazino-nicotinamide ligand, as a nicotinic acid analogue, is a bifunctional chelator capable of binding to the NH_2_-terminal amino acid and lysine residues of proteins on the one hand and of sequestering ^99m^Tc on the other. Using tricine as co-ligand, the Hynic-Annexin V conjugate proved a most stable complex and allowed fast and efficient labelling with ^99m^Tc in the presence of stannous ions. The one-step reaction provides ^99m^Tc-HYNIC-Annexin V in high radiochemical yields of typically 92-95 % without any additional purification step, with very high specific activities of 198-265 GBq/μmol. The radiolabeling procedure was further improved into a kit formulation of two vials requiring only 15 min of reaction (^99m^Tc-HYNIC-Annexin V, Theseus Imaging Corporation, Boston, USA). Compared with ^99m^Tc-BTAP-Annexin V, the formulation of ^99m^Tc-HYNIC-Annexin V offers a much simpler and faster preparation with a significantly higher radiochemical yields at room temperature, and requires substantially lower start activities (1.11-1.48 GBq). All these advantages make ^99m^Tc-HYNIC-Annexin V much more suitable for routine production and fast application in a clinical setting. In the follow-up of ^99m^Tc-BTAP-Annexin V, ^99m^Tc-(N-1-imino-4-mercaptobutyl)-Annexin V (^99m^Tc-i-Annexin V), a third type of ^99m^Tc-Annexin V, was developed and investigated soon afterwards in human subjects [56, 57]. To achieve labeling of acceptable quality, ^99m^Tc-i-Annexin V was prepared by incubating ^99m^TcO_4_^−^ with (N-1-imino-mercaptobutyl)-Annexin V (Mallinckrodt, Petten, The Netherlands) in the presence of stannous ions for 2 h at room temperature. Radiolabelling of Annexin V yielded ^99m^Tc-i-Annexin V with a radiochemical purity of 79 ± 3 %. However, the radiolabeling efficiency decreased from 83 % to 76 % during the shelf life of the radiopharmaceutical kit. These problems make it less suitable for common use in clinical settings.

Biodistribution and dosimetry. In a phase I study, eight patients with normal kidney and liver functions were employed to investigate the biodistribution and dosimetry of ^99m^Tc-BTAP-Annexin V [58]. It was found that predominant radioactivity accumulated in the kidneys, liver (28 ± 8 %ID and 20 ± 4 %ID at 70 min p.i., respectively) and urine bladder over time (approximately 73 %ID). However, the fast and extensive bowel excretion may preclude its clinical use for apoptosis imaging of the abdomen region. The biological half-life of the activity in the total body was 16 ± 7 h. The high absorbed dose to the kidneys, urinary bladder and spleen was 63 ± 22 μGy/MBq, 20 ± 6 μGy/MBq, 15 ± 3 μGy/MBq. The effective dose for the average injected activity of 600 MBq was determined to be 7.6 ± 0.5 μSv/MBq, or 4.6 ± 0.3 mSv. The first clinical trial with ^99m^Tc-HYNIC-Annexin V were performed to investigate the safety and to quantify its biodistribution and radiation dose [59]. Similar to ^99m^Tc-BTAP-Annexin V, ^99m^Tc-HYNIC-Annexin V showed a high accumulation in the kidneys (49.7 ± 8.1 %ID at 3 h p.i.), liver (13.1 ± 1.0 % ID at 3 h p.i.), and urine bladder over time (22.5 ± 3.5 %ID at 24 h p.i.). Excretion of the activity was almost exclusively through the urine, with a biologic half-life of 69 ± 7 h. The favorable biodistribution of ^99m^Tc-HYNIC-Annexin V may allow imaging of apoptosis in the abdominal as well as thoracic region. From a dosimetry point of view, the organs that receive high absorbed dose are kidneys, spleen and liver with a dose of 196 ± 31 μGy/MBq, 41 ± 12 μGy/MBq, 16.9 ± 1.3 μGy/MBq, respectively. The effective dose was 11.0 ± 0.8 μSv/MBq, corresponding to a total effective dose of 2.8 ± 0.2 mSv for a nominal injected activity of 250 MBq. In a short conclusion, ^99m^Tc-HYNIC-Annexin V is a safe radiopharmaceutical with a favorable biodistribution for imaging of apoptosis nearly everywhere in the body with an acceptable radiation dose. The phase I study of ^99m^Tc-i-Annexin V included six patients with myocardial infarction, one with Crohn's disease, and one healthy volunteer [56]. The radiopharmaceutical strongly accumulated in the kidneys (21 ± 6 %ID at 4 h p.i.) and to a lesser degree in the liver (12.8 ± 2.2 %ID at 4 h p.i.). ^99m^Tc-i-Annexin V generally showed predominantly urinary excretion with a biologic half-life of 62 ± 13 h. The high absorbed doses were found to be (93 ± 24 μGy/MBq for kidneys, 22 ± 6 μGy/MBq for spleen and 17 ± 2 μGy/MBq for liver. The effective dose was 9.7 ± 1.0 μSv/MBq, or 5.8 ± 0.6 mSv for a nominally injected activity of 600 MBq. From a dosimetric point of view, ^99m^Tc-i-Annexin V is therefore well suited for the study of apoptosis in patients.

Phase I/II and II/III clinical trials. Over the past years, a number of phase I/II and II/III clinical trials have confirmed the capability of ^99m^Tc-Annexin V radiopharmaceuticals for localization of apoptosis at sites of diseases in humans and have therefore established the crucial value of these radiopharmaceuticals as *in vivo* imaging biomarker of apoptosis. Increasing single site or even multicenter studies are still ongoing to get more clinical experience in this emerging field. In addition, systematic review and meta-analysis of these clinical imaging trials also highlight the reliability as well as the reproducibility of ^99m^Tc-Annexin V for imaging of apoptosis in humans [60–62].

**Figure 1:**
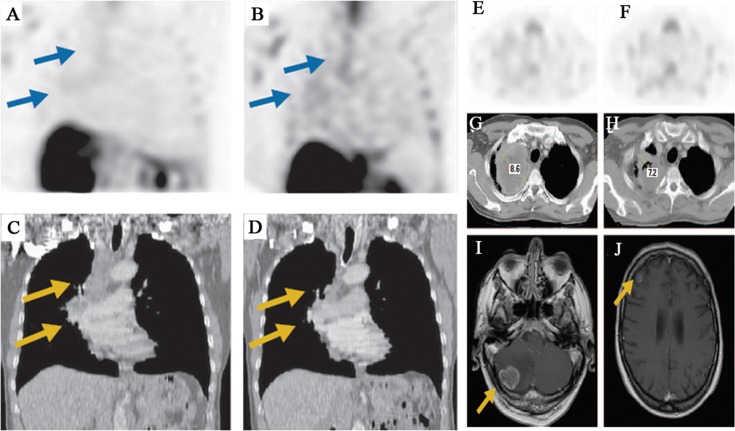
^99^mTc-Annexin V SPECT imaging of tumor response during platinum-based chemotherapy in advanced lung cancer **A**. Baseline and **B**. follow-up ^99m^Tc-Annexin V imaging demonstrate chemotherapy-induced increase of tumor tracer uptake (arrows). **C**. Baseline computed tomography demonstrates solid mass in the right upper lobe enlarged mediastinal lymph nodes (arrows). **D**. Follow-up computed tomography scan 8 weeks after the start of chemotherapy shows complete response (arrows). **E**. Baseline and **F**. follow-up ^99m^Tc-Annexin V imaging demonstrate therapy-induced decrease of Annexin V uptake. **G**. Baseline computed tomography shows heterogeneous mass in the right upper lobe. Follow-up **H**. computed tomography and magnetic resonance scans obtained 4 weeks later demonstrate local stable disease and brain metastases (arrows) in the **I**. cerebellum and **J**. right frontoparietal area.

In oncology trials, ^99m^Tc-Annexin V could provide insight into the biology and native apoptosis in tumors. The study in 18 patients with head and neck cancer showed a well correlation between the quantitative ^99m^Tc-Annexin V tumor uptake and the number of apoptotic tumor cells derived from TUNEL assays. However, mean percentage absolute tumor uptake of the injected dose per cm^3^ tumor volume was only 0.0003 % at 1 hour post-injection and 0.0001 % at 5 to 6 hours (*P* = 0.012) [23]. In another study in intracardiac tumor patient, it indicated that, as the high apoptotic index is found frequently in malignant but not in benign tumors, ^99m^Tc-Annexin V imaging may be helpful to study tumor biology in a noninvasive way which is difficult to access for biopsies [24]. Overall, these results have demonstrated that ^99m^Tc-Annexin V has the capability for binding to apoptotic tumor cells and the ability to study tumor biology in patients. Other studies have been proposed to evaluate its clinical feasibility for imaging apoptosis following cancer therapy to predict response. ^99m^Tc-Annexin V imaging has been used to image patients with breast cancer, lung cancer, lymphoma, head and neck cancer, leukemia, and soft tissue sarcomas treated with varying types of chemotherapy and/or radiation therapy. The ^99m^Tc-Annexin V uptake was correlated well with either Response Evaluation Criteria In Solid Tumors (RECIST) criteria or with cytological and pathological gold standards. In a study of 38 patients with different tumor types (non-Hodgkin lymphoma (NHL) n = 31; non-small-cell lung carcinoma (NSCLC) n = 4; head and neck squamous cell carcinomas n = 3) treated with various modalities of therapy, ^99m^Tc-Annexin V scintigraphy was acquired before and early after the start of treatment [27]. The changes in ^99m^Tc-Annexin V tumor uptake after therapy were visually and quantitatively calculated and correlated to tumor response according to RECIST criteria. A statistically highly significant correlation was found between changes in ^99m^Tc-Annexin V tumor uptake and clinical response (r^2^ = 0.62; *P* < 0.0001). Similarly, in a phase II/III study ^99m^Tc-Annexin V scintigraphy was performed to evaluate the early response of platinum-based chemotherapy before and within 48 hours after the start of therapy in 16 patients with advanced NSCLC. It showed a significant correlation between the ^99m^Tc-Annexin V tumor uptake and treatment outcome (r^2^ = 0.86; *P* = 0.0001). As shown in Figure 1, patients with notably increased Annexin V tumor uptake showed complete or partial remission while patients with stable or progressive disease demonstrated less prominently increased or decreased uptake of ^99m^Tc-Annexin V [28]. Collectively, these studies indicate that ^99m^Tc-Annexin V scintigraphy might be valuable as a predictive test for early therapy response during the process of treatment. Table 2 summarizes other clinical trials with ^99m^Tc-Annexin V imaging in oncology. In the field of nuclear medicine, ^18^F-FDG PET imaging has been considered as the gold standard for evaluation of oncology patients [75]. However, the radiopharmaceutical ^18^F-FDG could not specifically image apoptosis induced by chemotherapy or radiation therapy. Confounding imaging patterns such as reactive inflammation *versus* residual tumor may occur in ^18^F-FDG PET imaging [76]. Nevertheless, imaging of apoptosis with ^99m^Tc-Annexin V, immediately before and after therapy, may complement the deficiency in ^18^F-FDG PET imaging of malignancies in time [50].

**Table 2:** Clinical trials with 99mTc-Annexin V SPECT imaging in oncology

Authors	Study Type	Clinical Modals	Detection Rate (Sentivity)	Ref
Belhocine *et al*.	Phase I/II (n = 15)	NSCLC, SCLC, BC, NHL, HL treated by chemotherapy	7/15(46%) Increased uptake in primary or metastatic tumor	[63]
Vermeersch *et al*.	Phase I/II (n = 18)	primary HNC treated by surgery	11/18(61%) Identification of primary lesions and lymph node	[64]
Rottey *et al*.	Phase I/II (n = 20)	BC, MM, HNC, SCLC treated by chemotherapy	16/17(94%) A 25% change threshold of the ratios of tumor activity to background activity	[65]
Rottey *et al*.	Phase I/II (n = 23)	SCCHN, BC, RCC treated by radio-/ chemotherapy	12/23(52%) T/N ratio Median 2.5, range 0.3-4.2	[66]
Rottey *et al*.	Phase I/II (n = 11)	SCCHN, BC, RCC, Ovarian cancer, Bladder cancer	No significant differences in mean percentages of uptake between treated and control group	[67]
Kartachova *et al*.	Phase I/II (n = 33)	Lymphoma, NSCLC, SCCHN treated by radio-/chemotherapy	22/29(76%) A marked increase of uptake associated with complete or partial tumor response	[68]
Haas *et al*.	Phase I/II (n = 11)	Follicular lymphoma treated by radiotherapy	10/11(91%) Increased tumor uptake after 2 × 2 Gy involved field radiotherapy	[69]
Hoebers *et al*.	Phase III (n = 16)	Advanced HNSCC treated by chemoradiation	24/26 parotid glands (92%) Aradiation-dose-dependent uptake within dose range of 0-8 Gy	[70]
Loose *et al*.	Phase I/II (n = 29)	SCCHN treated by radiotherapy	Median T/N ratio: 2, range 1.0-5.1; Inverse correlation between T/N ratio and survival	[71]
Vermeersch *et al*.	Phase I/II (n = 13)	squamous head and neck carcinomas	Mean of difference for intra-, inter-, and day-to-day measurements: -3.4%, 2.4%, and -6%, respectively	[72]
Vermeersch *et al*.	Phase I/II (n = 28)	Primary and locally recurrent SCCHN	The absolute uptake related to MVD, MMP-9 and FasL expression as well as tumor-infiltrating lymphocytes	[73]
Kurihara *et al*.	Phase I/II (n = 10)	Breast cancer	9/10(90%) Higher values of T/N ratios	[74]

Apoptosis plays a dominant role in typical pathologic processes of cardiovascular disease such as myocardial infarction, heart failure, and atherosclerosis. Clinical trials with ^99m^Tc-Annexin V imaging in patients with cardiovascular disease have therefore been implemented, as shown in Table 3. Imaging of apoptosis with ^99m^Tc-Annexin V as well as perfusion with ^99m^Tc-sestamibi, ^99m^Tc-tetrofosmin, or ^201^Tl has been carried out in myocardial infarction patients with reperfusion obtained by percutaneous transluminal coronary angioplasty (PTCA) [29, 30]. The increased uptake of ^99m^Tc-Annexin V at the infarct sites strongly correlated with areas of myocardial hypoperfusion, which indicates the presence of myocardial apoptosis in the infarct area. Furthermore, corresponding to the decrease in ^99m^Tc-sestamibi defect size, the uptake of ^99m^Tc-Annexin V in the infarcted area was absent 4 d after acute myocardial infarction, which indicates that in parts of the area at risk, reversible myocardial damage rather than necrosis is present in cardiomyocytes [30]. A second application of molecular imaging of apoptosis with ^99m^Tc-Annexin V was first described in 2001 by Narula *et al*. on cardiac allograft rejection in transplant patients [36]. In the present study of 18 cardiac allograft recipients, 13 patients had negative and five had positive myocardial uptake of ^99m^Tc-Annexin V. These latter five also demonstrated transplant rejection and caspase-3 activation in endomyocardial biopsy, which remains the gold standard for diagnosis of cardiac allograft rejection in clinic. Another study with ^99m^Tc-Annexin V imaging as a noninvasive monitoring of acute rejection in heart transplantation at Stanford University Medical Center also showed that histological evidences of rejection was found in patients with increased uptake of the radiopharmaceutical [37]. These two studies reveal the clinical feasibility and safety of ^99m^Tc-Annexin V imaging for noninvasive detection of transplant rejection. In addition, ^99m^Tc-Annexin V imaging has been investigated as a new option in patients with other cardiovascular disease. For example, Kietselaer *et al*. has proven the feasibility of ^99m^Tc-Annexin V imaging in 9 patients with severe congestive heart failure (left ventricular function, <0.35) [31]. The myocardial uptake of this radiopharmaceutical strongly correlated with worsening of the disease. Imaging of phosphatidylserine expression in unstable plaques is also possible. In patients with atherosclerotic lesions in the carotid artery, the ^99m^Tc-Annexin V uptake in the lesions highly correlated with plaque instability [32]. This pilot study indicated that ^99m^Tc-Annexin V imaging may help to identify plaque instability. Despite the great success, whether ^99m^Tc-Annexin V imaging can be used as a diagnostic tool for cardiovascular disease in the human situation is as yet unknown. Investigations involving more patients are ongoing to establish in this area.

**Table 3:** Clinical trials with 99mTc-Annexin V SPECT imaging in cardiovascular and other diseases

Authors	Study Type	Clinical Modals	Detection Rate (Sentivity)	Ref
Thimister *et al*.	Phase I/II (n = 9)	Myocardial infarction	9/9(100%) Increased uptake; absent uptake in 2 patients 1-3 wk after the MI onset	[29]
Hofstra *et al*.	Phase I/II (n = 7)	Myocardial infarction	6/7(86%) Increased uptake	[30]
Narula *et al*.	Phase I/II (n = 18)	Cardiac allograft rejection	5/18(28%) Non-diffuse uptake	[36]
Kown *et al*.	Phase I/II (n = 10)	Cardiac allograft rejection	2/10(20%) Multifocal uptake	[37]
Kietselaer *et al*.	Phase I/II (n = 9)	Heart failure	5/9(56%) Multifocal uptake	[31]
Kietselaer *et* a*l*.	Phase I/II (n = 4)	Atherosclerosis	2/4(50%) Uptake in unstable plaque	[32]
Lorberboym *et al*.	Phase I/II (n = 12)	Acute stroke	7/7(100%) Abnormal increased uptake correlated with BBB permeability	[33]
Blankenberg *et al*.	Phase I/II (n = 2)	Acute stroke treated by neuroprotective therapy	2/2(100%) Multifocal uptake	[34]
Lampl *et al*.	Phase I/II (n = 12)	AD and non-AD dementia	4/5(80%) Abnormal foci of increased uptake	[35]
Lorberboym *et al*.	Phase I/II (n = 7)	Infection of prostheses	4/7(57%) Focal or linear uptake	[40]
Brande *et al*.	Phase I/II (n = 14)	Crohn's disease treated by infliximab	10/14(71%) A mean increase of 98.7% in colonic uptake	[38]

In neurology trials, there have been clinical trials of ^99m^Tc-Annexin V for imaging patients with acute stroke or dementia in which apoptosis may play a vital role (Table 3) [33–35]. In patients with acute stroke, the pilot image data showed the ability of ^99m^Tc-Annexin V to concentrate at sites of ischemic injury, which correlates to sites of restricted diffusion on MR imaging. Furthermore, compared with MR imaging, the patterns of ^99m^Tc-Annexin V uptake is heterogeneous and multifocal. This implied that ^99m^Tc-Annexin V imaging may better reflect sites of varying degrees of ischemic injury than MR imaging on a molecular level. Moreover, the uptake of ^99m^Tc-Annexin V in these patients coincided with the degree of blood-brain barrier (BBB) permeability [33]. In the study of Lampl *et al*., four of five patients with Alzheimer dementia (AD) showed multifocal cortical ^99m^Tc-Annexin V uptake, whereas patients with non-AD dementia and non-diseased healthy age-matched controls had normal SPECT image, which suggested the feasibility of ^99m^Tc-Annexin V for imaging AD.

**Figure 2:**
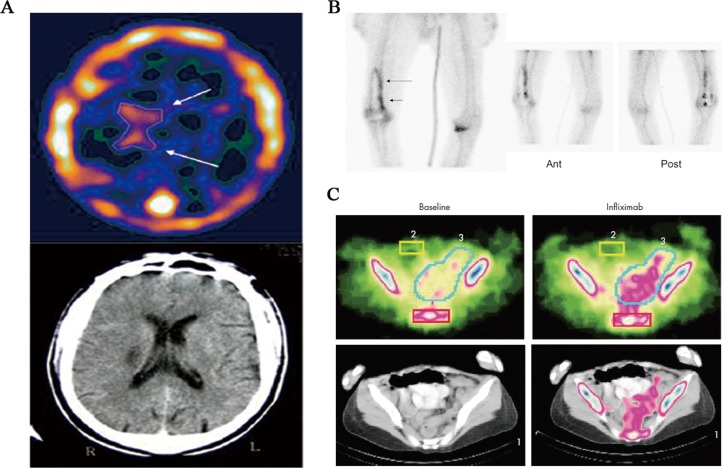
^99^mTc-Annexin V SPECT imaging in patients with stroke, infection of prostheses, and Crohn's disease **A**. ^99m^Tc-Annexin V brain SPECT imaging of a patient with a right peri-ventricular stroke, showing a wider distribution of Annexin V (top, arrows) compared to the CT findings (bottom). **B**. ^99m^Tc-Annexin V SPECT imaging of unilateral right knee prosthesis and signs of loosening on plain radiograph. Linear increased activity around prosthesis on bone scan (planar image far left; arrows) is demonstrated, with similar activity noted on anterior (Ant) and posterior (Post) views of Annexin V study (planar images; center and right). **C**. Example of ^99m^Tc-Annexin V SPECT imaging of human Crohn's disease. Apoptosis in the intestine of patients with Crohn's disease was visualised just before (baseline) and 24 h after infliximab treatment. The scintigraphic signals obtained corresponded with the diseased regions in the colon, indicating a correlation with disease localisation and increased ^99m^Tc-Annexin V uptake.

In addition, as shown in Table 3, ^99m^Tc-Annexin V radiopharmaceuticals have also been used to image patients with diseases such as infection of prostheses, Crohn's disease, glaucoma [38–40]. Preliminary clinical data in these patients demonstrated the clinical use of ^99m^Tc-Annexin V as a diagnostic biomarker for monitoring apoptosis (Figure 2).

Although all these results are encouraging, the wide clinical use of ^99m^Tc-Annexin V imaging remains a challenge. This was most likely because the specificity of ^99m^Tc-Annexin V for apoptotic cells (i.e. cannot discriminate apoptosis and necrosis in certain cases) and imaging sensitivity of SPECT were suboptimal [50, 77]. The limitations of ^99m^Tc-Annexin V SPECT imaging have motivated a search for PET radiopharmaceuticals for molecular imaging of apoptosis in humans.

### ^18^F-ML-10 PET imaging of apoptosis

The apoptotic membrane imprint including a complex of cellular alterations in plasma membrane potential, external plasma membrane leaflet and cytosol, phospholipid scrambling with preservation of membrane integrity takes place early in the process of apoptosis, which is one of hallmarks of apoptotic cells [20, 78]. Accordingly, a set of novel small-molecule probes that comprise the ApoSense family such as DCC, NST-732 and dansyl-ML-10 have been rationally designed to detect the concerted concurrence of this complex cellular alterations through selective accumulation within apoptotic cells driven by the irreversible membrane depolarization, cellular acidification, and apoptotic scramblase activation, and have been performed *in vivo* [79, 80]. In addition, Zeng and his coworkers have developed DFNSH and DNSBA molecules that are derivative of the Aposense compound NST-732 for imaging of apoptosis [81, 82]. Among them, ^18^F-ML-10 is the first PET radiopharmaceutical for molecular imaging of apoptosis that has advanced into the clinical stage of development, with promising results to date in several small scale clinical trials.

Radiosynthesis. ^18^F-ML-10 comprises a compact structure (molecular weight, 206), a minimal number of functional groups, and an ^18^F radioisotope, making it capable of meeting the challenge of clinical imaging of apoptosis by PET [83]. This minimized structure is amphipathic, designed to target this unique apoptosis-related complex of cellular alterations, cross the cell membrane, and accumulate in the cytoplasm, while it preserves the functional groups of its family for detection of apoptosis. ^18^F-ML-10 was synthesized by nucleophilic substitution from its respective precursor ML-10-mesylate on a widely available automated module GE TRACERLab^®^ FX-FN with a decay corrected yield of 39.8 % ± 8.4 % (n = 7) in 70 min and a specific activity of 235 ± 85 GBq/μmol at the end of synthesis and passed the quality control in a clinical GMP environment, which is commonly requested in clinical PET centers [84].

Biodistribution and dosimetry. In a phase I trial on healthy volunteers, ^18^F-ML-10 was highly stable *in vivo* and exhibited favorable biodistribution profiles followed by rapid clearance, with an elimination half-life of 1.3 ± 0.1 h from the blood and 1.1 ± 0.2 h from all other organs, and excretion through the urine [85]. The average effective whole-body dose was determined to be 15.4 ± 3.7 μSv/MBq, which is comparable to a clinical ^18^F-FDG PET scan (16-18 μSv/MBq). The urinary bladder received the highest dose of activity, and was therefore the dose-limiting organ. When voiding frequency was changed from once every 3 h to once every hour, the dose to urinary bladder was decreased by a factor of 2.8. In addition, all male subjects showed selective accumulation and retention of ^18^F-ML-10 in the testes. Administration of this radiopharmaceutical was safe, without adverse effects. These data support further development of this small molecule radiopharmaceutical for clinical PET imaging of apoptosis.

**Figure 3:**
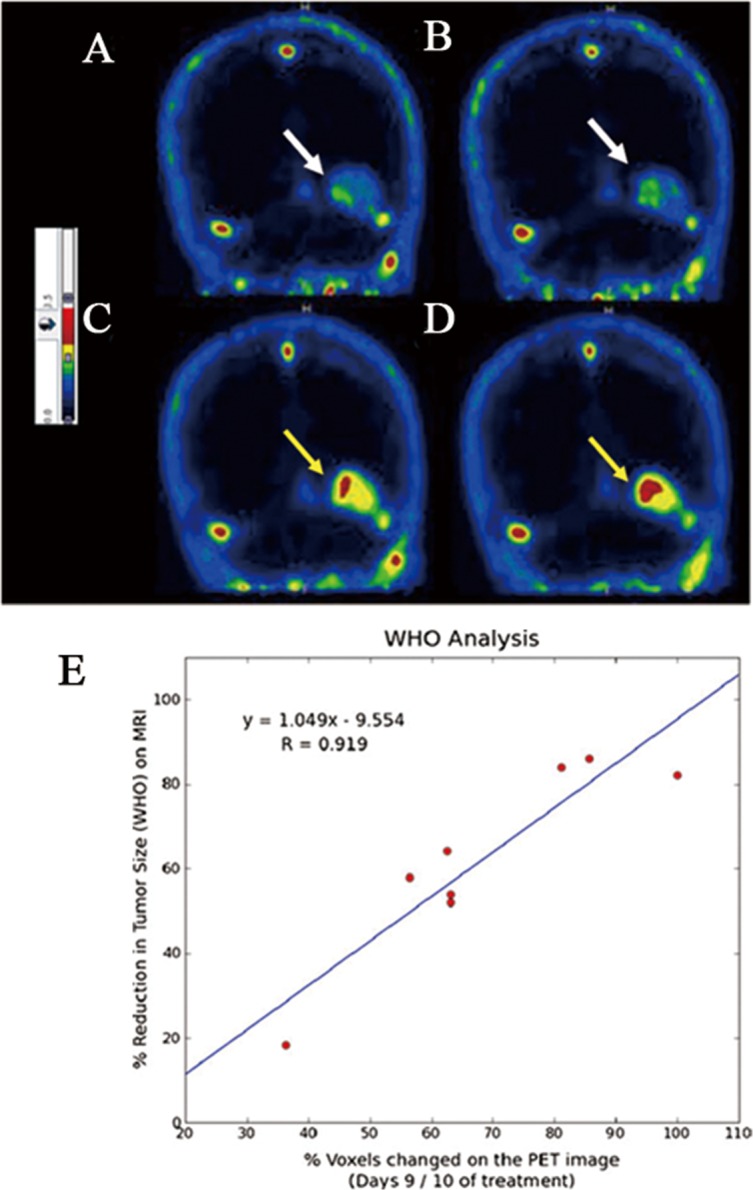
Assessment of response of brain metastases to radiotherapy by PET imaging of apoptosis with 18F-ML-10. ^18^F-ML-10 uptake is seen on the baseline PET scan **A**., **B**. white arrows) and after ten fractions of radiation (C, D yellow arrows). All sections are normalized to the blood and evaluated by a common color-coded scale (PMOD/QT21). While the scans show signal at baseline (A, B) reflecting the basal apoptotic load, the corresponding regions after treatment **C**., **D**. show increased uptake, reflecting the apoptosis induced in the tumor by the radiation. Notable is the heterogeneity of the signal intensity in the tumor. **E**. Correlation between early changes in ^18^F-ML-10 uptake and the anatomic response evidence obtained by MRI following the WHO criteria 6-8 weeks after completion of therapy. The Pearson correlation coefficient was found to be very high (r = 0.919, *p* < 0.001).

Phase II clinical trials. In a phase IIa study, PET imaging with the radiopharmaceutical ^18^F-ML-10 has been achieved in patients with acute ischemic cerebral stroke [20]. Tridimensional presentation of summed PET image was obtained after administration of ^18^F-ML-10 (day 3 after onset). Various intensities of apoptotic cell death were seen within region of infarct. In patients with brain metastases treated with whole-brain radiation therapy, the increased uptake of ^18^F-ML-10 was shown on the PET scans and heterogeneity of signal intensity was observed within the ^18^F-ML-10 hot spots (Figure 3). Voxel-based analysis of a mean change in ^18^F-ML-10 tumor uptake in the volume of interest (VOI) was determined to be 69.9 %, ranging from 36.3 % to 100 %, which highly correlated with the anatomic response evidence obtained by MRI following the WHO criteria 6-8 weeks after completion of therapy (r = 0.919, *p* < 0.001) [41]. These studies show the potential of ^18^F-ML-10 as a PET radiopharmaceutical for clinical imaging of apoptosis in tumors. Ongoing international multicenter studies in multiple tumor types are now being performed.

**Figure 4:**
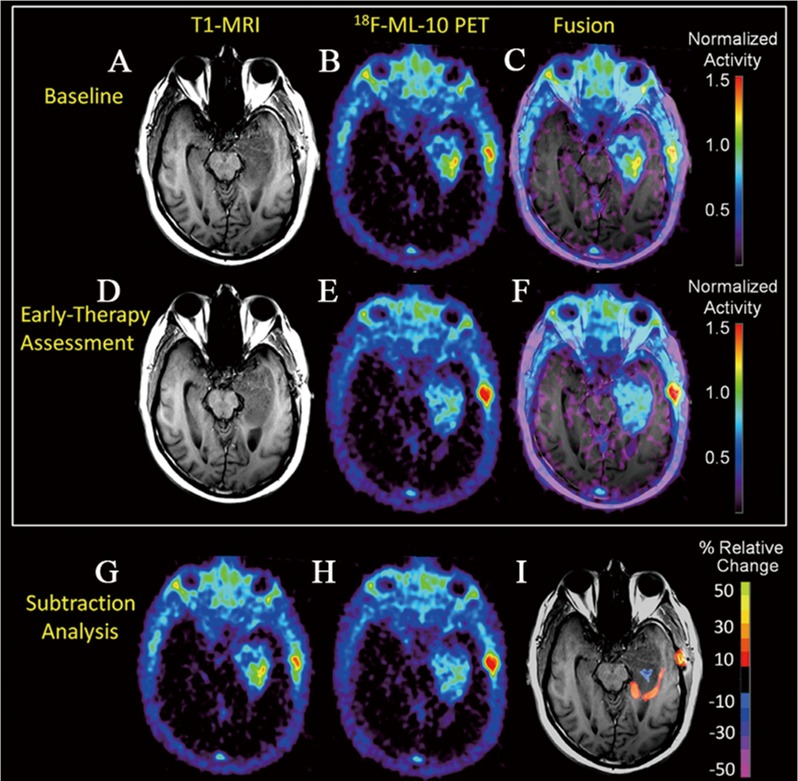
The use of ^18^F-ML-10 PET to assess apoptosis change in a newly diagnosed GBM patient before and early after therapy At baseline, the subject's T1-MRI **A**. shows left temporal lobe GBM. ^18^F-ML-10 uptake at baseline PET **B**. shows a region of high tracer uptake corresponding to the site of the GBM on baseline T1-MRI as seen in the PET-MRI fusion image **C**. After 3-weeks of therapy, the subject received a new set of T1-MRI **D**. and ^18^F-ML-10 PET **E**. images to assess for response. **F**. shows the T1-MRI and ^18^F-ML-10 PET fusion image. Normalized voxel-by-voxel subtraction cluster map of baseline (B and G) from PET (E and H) is shown fused to T1-MRI. Regions of the GBM exhibiting high baseline ^18^F-ML-10 uptake show reduced uptake at early-therapy assessment time-points (blue), while new regions (compared to baseline) of ^18^F-ML-10 uptake are observed at the tumor periphery (red/orange).

However, the precise mechanism of this radiopharmaceutical targeting the apoptotic cell membrane is unclear. In addition, one should keep in mind that malignant tumors often destroy the permeability of blood-brain barrier (BBB), which potentially causes excessive accumulation of radiopharmaceutical in tumors, thereby giving a higher baseline uptake before therapy compared to tumors without BBB alteration [25]. This explanation is supported by a recent study in which PET imaging with ^18^F-labeled ML-10 was used to evaluate changes of apoptosis in a newly diagnosed glioblastoma multiform (GBM) patient before and early after therapy [42]. As shown in Figure 4, at the baseline and early-therapy assessment time point, the uptake of ^18^F-ML-10 was observed at the site of the GBM, which corresponded to the GBM anatomical location on MRI scans. Moreover, a different pattern of ^18^F-ML-10 distribution was acquired at early-therapy assessment time point compared to the baseline. Normalized pixel-by-pixel subtraction analysis quantitatively demonstrated reduction in radiopharmaceutical uptake at the site of greatest baseline uptake, but increased uptake around the periphery of the tumor at early-therapy assessment time point. Therefore, further study is necessary to validate the specificity of ^18^F-ML-10.

### ^18^F-CP18 PET imaging of apoptosis

Caspases are a family of intracellular cysteine proteases that are responsible for the initiation and execution of apoptosis [86]. To date, there are 14 caspases identified and divided into two groups: initiator caspases (such as caspase-2, -8, -9, and -10) and effector caspases (caspase-3, -6, and -7) [87]. Although three different pathways have been identified for initiating apoptosis (i.e., the extrinsic and intrinsic pathways, endoplasmic reticulum stress pathway), all the pathways ultimately converge on the executive enzyme caspase-3/7 [88, 89]. Therefore, caspase-3/7 is an attractive marker for apoptosis. As a consequence, many types of caspase-3/7 inhibitors and substrates with different specificity have been constructed. Among these specific caspase-3/7 inhibitors and substrates, a subset of agents with a tag (radionuclide, fluorophore etc.) have been developed and are undergoing clinical translational research. For example, ^18^F-CP18 is the first substrate-based caspase-3/7 radiopharmaceutical for PET imaging of apoptosis in clinical trials.

**Figure 5:**
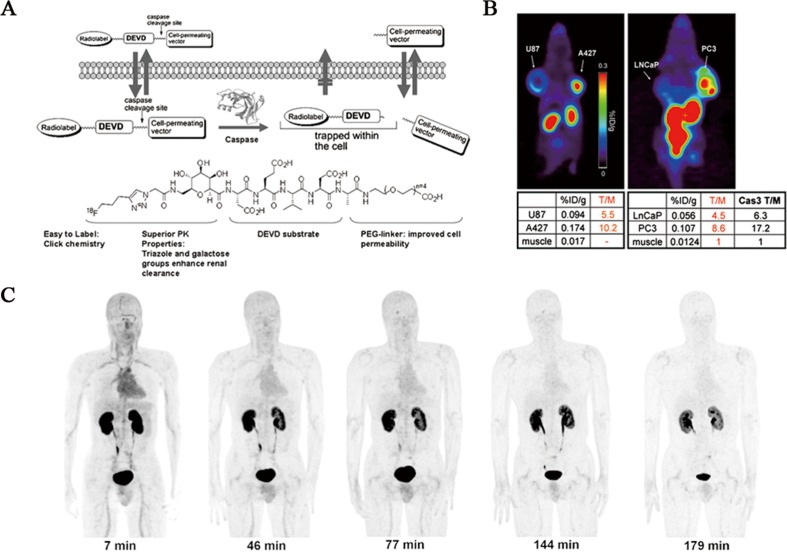
Evaluation of ^18^F-CP18 as a PET imaging tracer for apoptosis **A**. Design of caspase-3 substrate-based PET radiopharmaceutical ^18^F-CP18. **B**. *In vivo* ^18^F-CP18 PET imaging of dual-implant xenograft tumor-bearing mice. Preclinical studies showed caspase-3-dependent uptake of this radiopharmaceutical. **C**. Decay-corrected anterior maximum-intensity projections of PET at 7, 46, 77, 144, and 179 min (from left to right) after injection of ^18^F-CP18 in male volunteer. There was rapid clearance of activity in all organs.

Radiosynthesis. ^18^F-CP18 was designed as a substrate-based radiopharmaceutical for imaging of caspase-3/7 activity [90, 91]. From the standpoint of chemical structure, ^18^F-CP18 comprises a tetrapeptidic caspase substrate sequence “D-E-V-D” (Asp-Glu-Val-Asp) as recognition element, a polyethylene glycol (PEG) chain and galactose moiety to facilitate transport across cell membranes and maintain optimal pharmacokinetic properties and an ^18^F radioisotope for PET imaging of apoptosis (Figure 5A). Mechanistically, the cell permeating PEG fragment facilitates internalization of this radiopharmaceutical into cells undergoing cleavage in the presence of activated caspase-3/7. This cleavage step results in the preferential retention of radiolabeled moiety within cells as a function of caspase-3/7 activity. As more radiopharmaceutical is recognized and cleaved, more labeled fragments accumulate inside the apoptotic cells, resulting in signal enhancement. The ^18^F-CP18 was readily radiolabeled through the Cu(I)-catalyzed Huisgen reaction (“Click chemistry”) on an automated synthesis module (“Explora RN” from Siemens). Finally, this radiopharmaceutical was obtained in an average radiochemical yield of 40% and average specific activity of 175 GBq/μmol within 90 min.

Biodistribution and dosimetry. Preclinical studies showed caspase-3/7-dependent uptake of ^18^F-CP18 in apoptotic tumor cells, suggesting the potential of ^18^F-CP18 as a PET radiopharmaceutical for imaging apoptosis (Figure 5B). Soon after, biodistribution and radiation dosimetry of ^18^F-CP18 in healthy volunteers was investigated using whole-body PET/CT scans [43]. Relatively high activity uptake was observed in the kidneys and bladder with a mean standardized uptake values (SUVs) of 65 and 6, respectively at 50 min after injection, indicating a predominant renal clearance (Figure 5C). The liver and heart showed diffuse uptake with a SUV of 1.5 and 1.5, and the intestine regions have background levels activity during the period of imaging. The rapid clearance and relatively low nonspecific uptake of this radiopharmaceutical suggest a large imaging window available for imaging caspase-3/7 activity in tumors throughout the torso. The mean effective dose was 38 ± 4 μSv/MBq and 15 ± 2 μSv/MBq for the 4.8- and 1-h bladder voiding interval, with the urinary bladder wall having the highest absorbed dose at 536 ± 61 μGy/MBq and 142 ± 15 μGy/MBq, respectively. For a typical injected activity of 555 MBq, the effective dose for the 2 intervals would be 21.1 ± 2.2 mSv and 8.3 ± 1.1 mSv. From the radiation dosimetry perspective, because of receiving the highest radiation dose, the urinary bladder wall was deemed the critical organ. Both the effective dose and bladder dose could be decreased by bladder-voiding interval. In spite of reasonable biodistribution and dosimetry profiles of ^18^F-CP18 in human subjects, further studies are needed to evaluate the efficacy of this promising PET radiopharmaceutical. Currently, a phase II study (NCT01766622) of ^18^F-CP18 PET/CT imaging in patients with relapsed platinum resistant or refractory epithelial ovarian cancer, primary peritoneal cancer or fallopian tube cancer following treatment with Birinapant (a SMAC mimetic drug) has been undergoing and clinical imaging data has not been published yet.

**Figure 6:**
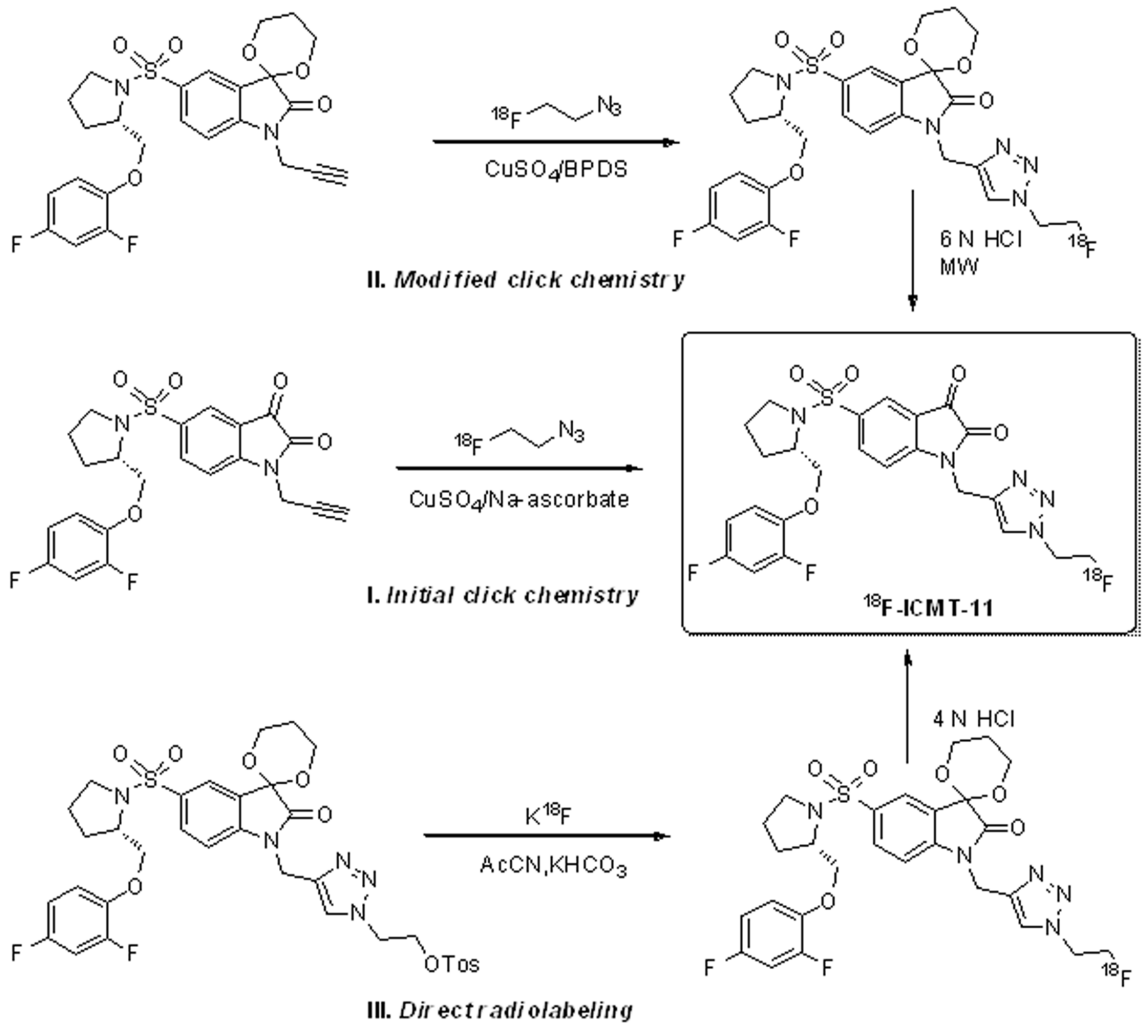
Radiochemistry development of ^18^F-ICMT-11

### ^18^F-ICMT-11 PET imaging of apoptosis

Although the initial research mainly focused on peptide-based substrates, these novel small-molecule caspase inhibitors, based mostly on the isatin sulfonamide moiety, have been advanced. Several inhibitors of this class have been labelled with either ^18^F or ^11^C radioisotope for PET imaging, including ^18^F-AF-110, ^18^F-ICMT-11, ^18^F-WC-II-89, ^11^C-WC-98, and ^18^F-WC-IV-3, which were evaluated in preclinical and clinical models [92–96]. Fortunately, ^18^F-ICMT-11 is the most promising imaging radiopharmaceutical of the isatin series and has been selected as a candidate by the QuIC-ConCePT consortium for clinical development.

Radiosynthesis. As shown in Figure 6, the radiosynthesis of ^18^F-ICMT-11 was originally achieved by a facile “click chemistry” stratagem using 2′-fluoroethyl-1,2,3-triazole precursor and 2-[^18^F]fluoroethylazide, resulting in modest specific activity of 1.2 GBq/μMol and a stable isatin analogue impurity at a concentration of 14 μg/mL [97]. In an effort to improve the specific activity of ^18^F-ICMT-11, a modified click radiochemistry strategy using acetal protection of the reactive isatin C-3 carbonyl as the 1,3-dioxane together with copper(I) stabilizing bathophenanthroline disulphonate (BPDS) as catalyst, was investigated. This led to ^18^F-ICMT-11 production in radiochemical yield of 3.0 ± 2.6 %, specific activity of 24 ± 19 GBq/μmol and stable isatin impurity concentration of 4.1 ± 4.1 μg/mL [98]. Further validation and clinical translation of this radiopharmaceutical, a robust and reliable automated radiosynthesis procedure using a protected tosylate precursor was developed and transferred onto a fully automated GE FASTlab synthesis platform under GMP conditions for further validation and optimization [99]. The automated radiosynthesis of ^18^F-ICMT-11 displayed a yield of 4.6 ± 0.4 GBq with a radiochemical purity of > 98 % and a specific activity of 685 ± 237 GBq/μmol within 90 min. Quality control has been implemented according to the European Pharmacopoeia, which demonstrated that this radiopharmaceutical can be consistently manufactured on the FASTlab to meet specifications. Finally, the automated procedure has been validated to clinically acceptable standards [100].

Biodistribution and dosimetry. In a phase I study, Challapalli and his coworkers reported the safety, biodistribution, and radiation dosimetry profiles of ^18^F-ICMT-11 in 8 healthy human volunteers [44]. This PET radiopharmaceutical was found to be safe and well tolerated in all subjects with no serious adverse events. The radioactivity of ^18^F-ICMT-11 was initially observed in the vascular and then rapidly distributed to the liver and kidneys, followed by elimination via the kidneys and hepatobiliary routes (Figure 9). In addition, the relatively high radioactivity within the gallbladder was detected, with slow washout into the gastrointestinal tract. The pattern of hepatic-based metabolism and gut clearance may limit abdominal imaging applications. The mean effective dose was estimated to be 0.025 ± 0.004 mSv/MBq (0.022 ± 0.004 mSv/MBq for men; 0.027 ± 0.004 mSv/MBq for women). The gallbladder wall, small intestine, upper large intestinal wall, urinary bladder wall and liver received the highest absorbed dose (0.59 ± 0.44, 0.12 ± 0.05, 0.08 ± 0.07, 0.08 ± 0.02, 0.07 ± 0.01 mGy/MBq, respectively). These results indicated ^18^F-ICMT-11 is a safe PET radiopharmaceutical with a favorable dosimetry profile for clinical imaging. Further clinical studies are now warranted to assess the utility of ^18^F-ICMT-11 in treatment-induced tumor apoptosis.

### Other radiopharmaceuticals

According to the modified IodoGen method, clinical grade ^123^I-Annexin V has been produced for human studies with radiochemical yields of 87.0 ± 6.5 %, radiochemical purities > 98 %, and average specific activities of 13400 MBq/μmol. Subsequently, the biodistribution and dosimetry of ^123^I-Annexin V in human volunteers was evaluated by Lahorte and his colleagues [45]. The acquired images with clinical grade radiopharmaceutical showed low lung uptake, making it suitable for imaging of thoracic regions, whereas delayed imaging revealed extensive bowel activity caused by dehalogenation of this radiopharmaceutical, resulting in the hamper of imaging in the abdominal region. The thyroid, kidneys, heart wall, liver and bone surfaces received the highest absorbed doses. On average, effective dose of ^123^I-Annexin V was estimated to be 0.02 mSv/MBq, which is acceptable to the patient for *in vivo* imaging. Furthermore, compared to ^99m^Tc labelled-Annexin V (6h), the advantageous longer half-life of ^123^I-Annexin V (13.2 h) allows imaging to be scheduled later after administration. Nevertheless, this radiopharmaceutical still needs more investigation in clinical studies. As yet, no more imaging studies have been described.

## CLINICAL IMPACT OF APOPTOSIS IMAGING

These ongoing translational research and clinical efforts have greatly increased our understanding of apoptosis and the clinical value of apoptosis imaging in pathogenesis and therapeutic regimens. In this regard, it is our opinion that a perspective of the impact of these clinical efforts is crucial to discuss and is necessary for a comprehensive review. Based on the publications, we categorize them into three strands of clinical impact, namely, apoptosis imaging as predictive and prognostic markers, early-response indicators and surrogate endpoints [101–104].

### Apoptosis imaging as predictive and prognostic markers

Molecular imaging of apoptosis as a predictive marker aims to indicate the course and progression of a disease, or objectively evaluate the likelihood of response to a specific clinical intervention. On the other hand, prognostic markers are a class of predictive markers that classify patient risk stratification and assess the patient's overall outcome independent of treatment. Moreover, in clinical practice, apoptosis imaging markers may have both predictive and prognostic value. Perhaps the clearest example of apoptosis imaging as predictive and prognostic markers is SPECT imaging with most widely performed radiopharmaceutical ^99m^Tc-Annexin V in oncology clinical trials. The ^99m^Tc-Annexin V uptake in tumors suggested a complete or a partial response, whereas no significant post-treatment tumor uptake indicated a progressive disease. Furthermore, the overall survival and progression-free survival were significantly relevant to the ^99m^Tc-Annexin V uptake in tumors after treatment. Although promising, more rigorous carefully controlled, and properly powered clinical trials need to be warranted for objectively establishing the positive and negative predictive value of ^99m^Tc-Annexin V with well-defined uptake thresholds, and supporting this indication. Increasingly, clinicians need to understand and interpret these markers in order to optimize clinical decision-making.

### Apoptosis imaging as an early-response indicator

The potential of ^18^F-FDG PET as an early indicator of response to therapy has been widely studied in oncology practice. Generally, a decrease in ^18^F-FDG uptake early during the course of chemotherapy is predictive of clinical outcome and is prognostic of cancers. However, it is also limited by overlap in between the tumor ^18^F-FDG uptake levels and different categories of response such as the uptake induced by treatment-related inflammation [76, 105, 106]. In practice, successful tumor treatments, such as radiation, chemotherapy, iatrogenically induces apoptosis. Molecular imaging of apoptosis may be even more robust and sensitive early-response indicators of therapy. The ^18^F-ML-10 PET imaging of apoptosis has been initially performed as an early-response indicator of brain metastases to whole-brain radiation therapy. Importantly, ^18^F-ML-10 PET could provide valuable information on response to treatment as early as 9 days after initial radiotherapy. Well-designed prospective studies are therefore needed to better explore this role for ^18^F-ML-10 PET imaging. In addition, early response to therapy has also been investigated in clinical trials with ^99m^Tc-Annexin V SPECT imaging. It is hoped that molecular imaging of apoptosis may serve as an early-response indicator for therapy in the clinical setting, and thereby assist in the transition to a more personalized approach in oncology.

### Apoptosis imaging as a surrogate endpoint

A surrogate endpoint is a marker for therapeutic success and reflects the desired therapeutic effect including disease-free survival, progression-free survival or overall survival [107]. Anatomic imaging has had success and is commonly used as a surrogate endpoint for treatment in clinical trials, most based on changes in tumor size according to the Response Evaluation Criteria in Solid Tumors (RECIST) criteria. However, size-based surrogate endpoints have some limitations, such as challenges for distinguishing residual tumor from adjacent normal tissues and significant inability to assess response to chemotherapy when anatomical changes in the tumor size is not observed [108]. Molecular imaging, especially ^18^F-FDG PET, is beginning to be used as an accepted surrogate endpoint for cancer therapy trials in era of RECIST 1.1 criteria [109]. Currently, it has not been accepted by the U.S. Food and Drug Administration as a surrogate endpoint for treatment outcome. As an emerging imaging modality, apoptosis imaging as a surrogate endpoint in clinical trials and clinical practice has been ongoing. The exciting initial study in patients with myocardial infarction showed no ^99m^Tc-Annexin V activity at the infarct site 4 d after PTCA, indicating that apoptotic cardiomyocytes has been rescued and regained their function. Although promising, whether molecular imaging of apoptosis can be used as a surrogate endpoint is as yet unknown. The next step is to accomplish further prospective studies to support more widespread acceptance of apoptosis imaging as a surrogate endpoint.

## CONCLUSIONS

During the past decade, great efforts have been made to develop radiopharmaceuticals for molecular imaging of apoptosis in clinical application. Following proof-of-concept studies with ^99m^Tc-Annexin V, several peptides and small molecules based radiopharmaceuticals have been developed to enable SPECT and PET imaging of apoptosis. Some of them like ^99m^Tc-Annexin V, ^18^F-ML-10, ^18^F-CP18, ^18^F-ICMT-11 have met the challenges required to qualify a radiopharmaceutical in clinical practice and are currently under clinical investigations on a wide scope of diseases. Although very promising, the integration of these radiopharmaceuticals into clinical practice still requires a concerted effort by all the involved parties including clinicians, oncologists, radiologists and pharmacologist. Additionally, the molecular imaging community also must come together, working with cooperative clinical trial groups and pharmaceutical industry, to support well-designed prospective trials validating apoptosis imaging as predictive and prognostic markers, early-response indicators and surrogate endpoints in clinical trials and clinical practice. In this way, one or more of these radiopharmaceuticals will ultimately progress to authorized approval and become widely used imaging agents in the clinic.
